# Ideal Standards, Acceptance, and Relationship Satisfaction: Latitudes of Differential Effects

**DOI:** 10.3389/fpsyg.2017.01691

**Published:** 2017-09-28

**Authors:** Asuman Buyukcan-Tetik, Lorne Campbell, Catrin Finkenauer, Johan C. Karremans, Gesa Kappen

**Affiliations:** ^1^Psychology Program, Sabanci University, Istanbul, Turkey; ^2^Department of Psychology, University of Western Ontario, London, ON, Canada; ^3^Interdisciplinary Social Science: Youth Studies, Utrecht University, Utrecht, Netherlands; ^4^EMGO Institute for Health and Care Research, VU University Amsterdam, Amsterdam, Netherlands; ^5^Department of Social Psychology, Radboud University Nijmegen, Nijmegen, Netherlands

**Keywords:** ideal standards, discrepancy, relationship satisfaction, partner acceptance, non-linear association

## Abstract

We examined whether the relations of consistency between ideal standards and perceptions of a current romantic partner with partner acceptance and relationship satisfaction level off, or decelerate, above a threshold. We tested our hypothesis using a 3-year longitudinal data set collected from heterosexual newlywed couples. We used two indicators of consistency: pattern correspondence (within-person correlation between ideal standards and perceived partner ratings) and mean-level match (difference between ideal standards score and perceived partner score). Our results revealed that pattern correspondence had no relation with partner acceptance, but a positive linear/exponential association with relationship satisfaction. Mean-level match had a significant positive association with actor’s acceptance and relationship satisfaction up to the point where perceived partner score equaled ideal standards score. Partner effects did not show a consistent pattern. The results suggest that the consistency between ideal standards and perceived partner attributes has a non-linear association with acceptance and relationship satisfaction, although the results were more conclusive for mean-level match.

## Introduction

Individuals possess ideal partner standards, or an idea of the traits and attributes they desire in a romantic partner (Fletcher et al., 1999). These standards have been reliably demonstrated to be associated with individuals’ evaluations of their relationship satisfaction. Specifically, greater consistency^1^ between ideal standards and perceptions of a current romantic partner is positively related to relationship satisfaction and psychological wellbeing (Fletcher et al., 1999; Campbell et al., 2001; Overall et al., 2006; Frost and Forrester, 2013).

It is unclear, however, how much of a consistency^2^ is optimal with respect to relationship evaluation, and how much of a discrepancy is acceptable. Although previous research suggested that maximum relationship satisfaction takes place at the highest levels of consistency (e.g., Fletcher et al., 1999), whether individuals could reach maximum satisfaction at lower levels of consistency is unexplored. Is the association between consistency and relationship satisfaction linear, such that progressively greater consistency predicts ever-increasing degrees of relationship satisfaction? Or is this association non-linear, with progressively greater consistency predicting greater relationship satisfaction up to a certain threshold, after which relationship satisfaction levels off? Our research goal is to determine if the form of the association between consistency and relationship satisfaction is non-linear, and if so identify the threshold above which the positive effects of greater consistency on partner acceptance and relationship satisfaction level off, or decelerate.

### Ideal Standards Model

The Ideal Standards Model (ISM; Fletcher et al., 1999, 2000) identified three main dimensions of standards. The first dimension includes standards about warmth, trustworthiness, and intimacy levels in a partner. The second dimension represents standards in the domain of passion in a relationship and attractiveness and vitality levels of a partner. The last dimension includes standards about a partner’s social status and resources. Although research showed that discrepancies between the ideals and partner’s attributes in the warmth/trustworthiness dimension are the most robust among the three dimensions when predicting relationship satisfaction, discrepancies along each dimension are associated with relationship satisfaction (Campbell et al., 2001).

### Latitudes of Differential Effects

How much of a consistency would be associated with the highest level of relationship satisfaction? It may be that each incremental increase in consistency (i.e., incrementally smaller discrepancies) would add to relationship satisfaction (i.e., linear association). Because individuals would aim to maximize their “utility” in their relationships, the perfect match between one’s ideal standards and partner attributes would bring the maximum satisfaction. Nevertheless, first, in the domain of a romantic relationship, most individuals may not have the opportunity to couple with a potential partner who can completely match their ideals. Even when this opportunity does occur, it may be difficult to maintain that relationship because that partner would be highly desired by others (Fletcher and Simpson, 2000; Simpson et al., 2001). Second, over their lives, individuals may learn that partners in general lack some qualities by observing their own and others’ relationships. Individuals therefore, may not expect their partner to possess all of their ideal standards (Baucom et al., 1989; Li and Fung, 2012). Individuals may develop over time and experience a threshold of an acceptable level of discrepancy between their ideal standards and partner attributes (cf. Kenrick et al., 2009; Fletcher et al., 2014). To illustrate, Kenrick et al. (2001) showed that a middle-level of income was enough for individuals to rate a potential marriage partner with a maximum level of desirability. After that level, however, any incremental increase in income did not make a significant contribution to the desirability of a potential partner.

In this study, we examine whether the relation of the consistency to partner acceptance and relationship satisfaction follows the so-called *diminishing returns principle*. That is, we investigate whether consistency is associated with partner acceptance and relationship satisfaction with a steep positive slope up to a threshold and whether the slope levels off at some high level of consistency. This hypothesis is in line with the Prospect Theory, which suggests that individuals are more sensitive to losses than they are to gains (Kahneman and Tversky, 1979; Kahneman, 1991). When an acceptable level of gain is reached, further gains may not add much value. Individuals, however, may evaluate each incremental increase in loss (i.e., a discrepancy from ideal standards) very negatively.

### How to Operationalize Consistency?

The most direct method in assessing the perceived consistency between ideal standards and perceptions of a current romantic partner is to ask participants to rate how much they believe that their partner matches their ideals on several attributes (Campbell et al., 2001; Overall et al., 2006). Instead of such a direct question, however, some studies (like our research) asked participants to rate both ideal standards and perceived partner attributes separately and used different methods to compute consistency (Fletcher and Kerr, 2010). For example, it is possible to examine the interaction effect between reported ideals and perceived partner ratings on the relationship quality (Eastwick and Neff, 2012). In such an approach, high and low levels are computed compared to the sample mean. For example, participants whose ideal standards scores are significantly higher than the other participants’ ideal standards score were considered as having high levels in ideal standards. This method, however, could not capture the operationalization of within-person consistency in the present study. For example, an individual’s reported ideals and perceived partner ratings can be high (i.e., higher than the sample mean), but the level of his perceived partner ratings can still be lower than the level of his ideal standards.

In this research, we used two indicators of consistency, which we labeled *pattern correspondence* and *mean-level match*, that are able to capture within-person comparison (i.e., one’s perceived partner attributes compared to one’s own ideal standards). *Pattern correspondence* was computed by calculating within-person correlations between ideal standards and perceived partner ratings, and could vary between -1 and 1. *Mean-level match* was computed by subtracting the average score across ideal standards from the average score across perceived partner ratings. Thus, negative and positive scores in this variable indicated that the perceived partner ratings fall short of and exceed ideal standards, respectively. These two indicators of consistency represent unique constructs (e.g., Epley and Dunning, 2006; Fletcher and Kerr, 2010). *Pattern correspondence* represents the consistency between the relative strengths of items across ideal standards and perceived partner ratings. *Mean-level match*, however, represents whether perceived partner ratings fall short of, or exceed ideal standards *on average* across all items. These two indicators do not always correlate with each other. For example, a participant might rate how much three standards (e.g., trustworthy, sexy, and ambitious) represent his ideal partner and give scores of 7, 5, and 3 (with a mean level of 5). His ratings for his current partner, however, could be 5, 7, and 3 (again with a mean level of 5), respectively. In this example, pattern correspondence shows a moderate level of consistency (i.e., *r* = 0.50), because trustworthiness is more important for him compared to sexiness, but he thinks that his partner is very sexy but not very trustworthy. His perception of his partner’s ambition level fits in with his ideals. Mean-level match, however, indicates a perfect consistency, because average of all three perceived partner ratings perfectly matches the average of three ideal standards (i.e., difference = 5–5 = 0).

Both pattern correspondence and mean-level match have been used in the literature and shown to be predictors of individual and relationship outcomes (e.g., relationship satisfaction, divorce, mental health). For example, Fletcher et al. (1999) used the pattern correspondence to operationalize consistency and showed its positive association with relationship quality (Study 6; see also Fletcher et al., 2000; Zentner, 2005; Eastwick and Neff, 2012 for the same method). Frost and Forrester (2013), however, preferred to use mean-level match to compute consistency, and revealed its links with relationship satisfaction, commitment, break-up thoughts and depression (see also Lee et al., 2008 for the same method). In this study, therefore, we used both of these consistency indicators and compared their results.

### Overview of the Study^3^

Using data from three waves of a longitudinal study among newlyweds, we hypothesized that the association between consistency and (1) partner acceptance and (2) relationship satisfaction is positive and linear up to a threshold, after which any incremental increase in consistency will be weakly predictive of partner acceptance and relationship satisfaction. Given that warmth/trustworthiness is typically the most influential dimension on relationship satisfaction (Campbell et al., 2001), we also proposed that individuals expect high levels of warmth/trustworthiness from their partner and thus will be less tolerable to larger discrepancies on this dimension compared to the other two dimensions (vitality and status). Last, we will examine the partner effects of consistency considering the finding that individuals’ relationship wellbeing is also affected by how much their partner perceives them to meet with their partner’s ideals (Sternberg and Barnes, 1985; Murray et al., 1996; Campbell et al., 2001). Similar to the actor effects, we expect that partner’s consistency (i.e., partner’s adherence to his/her expectations) will have a weaker association with actor’s acceptance and relationship satisfaction after a certain threshold than below that threshold.

## Materials and Methods^4^

### Participants and Procedure

Participants were heterosexual newlywed couples who participated in a five-wave longitudinal study on wellbeing and marriage in the Netherlands (for more information, see Finkenauer et al., 2009). Although there was no institutional ethics committee at VU University Amsterdam Faculty of Psychology and Education at the time of the first assessment (i.e., 2006), written informed consent forms were obtained from all participants before the data collection. The study was conducted in compliance with the regulations of the Netherlands Organization for Scientific Research, which funded the research project (NWO grant number: 452-05-322).

The data relevant to our research questions were collected at the second, third, and fourth study waves, which took place 1 year apart. There were 195 couples in the second wave. In the third and fourth waves, the number of participated couples decreased to 189 and 157, respectively. Average relationship duration at the second study wave was 6.51 years (*SD* = 3.05). Average ages were 32.91 (*SD* = 4.87) and 29.97 (*SD* = 4.25) for males and females, respectively.

### Measures

#### Ideal Standards

We assessed participants’ standards about their ideal partner using the modified versions of the ideal partner scales developed by Fletcher et al. (1999). The 16 standards used in this study were understanding, supportive, kind, good listener, sensitive, trustworthy, sexy, pays attention to his/her appearance, attractive appearance, good lover, adventurous, original, creative, inventive, successful, and ambitious. Participants rated how much each item represented their ideal partner using a 7-point Likert scale (1 = *not at all*, 7 = *very much*). Cronbach’s alpha levels ranged between 0.84 and 0.89 across three study waves (*M* = 0.87).

#### Perceived Partner Ratings

Using the same 16 ideal standards, participants reported the extent to which they believed their current partner possesses those attributes using a 7-point Likert scale (1 = *not at all*, 7 = *very much*). Cronbach’s alpha levels ranged between 0.85 and 0.88 across three study waves (*M* = 0.87).

#### Acceptance

To assess acceptance, we used the 6-item acceptance subscale of the Responsiveness Scale (Reis and Shaver, 1988; Birnbaum and Reis, 2006). Example items of the subscale were “I esteem my partner, shortcomings and all” and “I see the same virtues and faults in my partner as my partner sees in him/herself” (1 = *completely disagree* to 5 = *completely agree*). The range of Cronbach’s alpha levels across three study waves was between 0.69 and 0.77 (*M* = 0.73).

#### Relationship Satisfaction

We assessed relationship satisfaction using the Dyadic Adjustment Scale (Spanier, 1976). Sample items in our 30-item scale are “Do you confide in your partner?”; “How often do you think things are going well between you and your husband?” (1 = *never*, 5 = *all the time*). Cronbach’s alpha levels across three study waves ranged between 0.85 and 0.89 (*M* = 0.87).

## Results

### Strategy of Analysis

Our focus was on within-time associations, rather than longitudinal associations, of indicators of consistency (i.e., pattern correspondence and mean-level match) with acceptance and relationship satisfaction. Thus, we conducted our analysis using the over-time standard Actor-Partner Interdependence Model (APIM) (Kenny et al., 2006; Kashy and Donnellan, 2012). That is, we used all three study waves in the analyses and investigated the associations between the variables at the same time period, applying an autocorrelated covariance structure between the residuals at consecutive time periods to account for the interdependence between these ratings. In all models, we allowed men’s intercept, women’s intercept, and their covariance to vary across participants.

Regarding statistical power, it is difficult to provide an accurate estimate due to the dyadic longitudinal research design and the APIM analysis. During the data collection, efforts focused on recruiting around 200 couples at the fist assessment wave. Considering the facts that we had at least 157 couples at each wave and included all participants’ scores in our analysis, number of units at the upper level in our analysis was sufficient to have trustworthy estimates (i.e., more than 50 units; Maas and Hox, 2005). Additionally, over-time standard APIM analysis increased the robustness of our findings by providing us the advantage of examining the research questions using all three time periods in the data (Kashy and Donnellan, 2012). For example, we had 1,075 scores for ideal standards in our dataset (see **Table 1**). Last, Schönbrodt and Perugini (2013) showed that correlations stabilize when sample sizes approach 250. Given that our sample size (minimum number of scores per variable = 970, **Table 1**) is higher than 250, we believe that our sample size provided us adequate power for our analysis.

**Table 1:** Descriptive statistics and correlations among the study variables.

Variable	Minimum	Maximum	*M*	*SD*	*N*	1	2	3	4	5
(1) Ideal standards	2.38	7.00	5.73	0.53	1075	-				
(2) Perceived partner ratings	3.63	7.00	5.66	0.58	1079	0.61^∗∗^				
(3) Acceptance	1.50	5.00	4.19	0.44	1078	0.31^∗∗^	0.50^∗∗^			
(4) Relationship satisfaction	27.00	139.00	110.58	10.83	1080	0.28^∗∗^	0.55^∗∗^	0.52^∗∗^		
(5) Pattern correspondence	-0.49	1.00	0.47	0.30	970	-0.06	0.07^∗^	0.16^∗∗^	0.21^∗∗^	
(6) Mean-level match	-2.63	1.63	-0.08	0.50	1074	-0.36^∗∗^	0.53^∗∗^	0.26^∗∗^	0.34^∗∗^	0.15^∗∗^

*Descriptive statistics and correlations show the statistics across all three waves of data. ^∗∗^*p* < 0.001, ^∗^*p* < 0.05. *N* shows the number of scores included in the analysis. For example, the number 1075 for ideal standards represents the number of responses (number of couples across three study waves^∗^2 partners = 541^∗^2 = 1082) in the data minus the number of missing cases (*n* = 7) for that variable. *N* for pattern correspondence was low due to missing cases for variables used to compute this variable (*n* = 8) and having no variance in some participants’ ideal standards and/or perceived partner ratings (*n* = 104). No variance in those variables shows that the participant rated all items under one/both of these scales with the same score.*

To examine both linear and non-linear associations of pattern correspondence with acceptance and relationship satisfaction, we conducted analyses for linear, quadratic, cubic, and exponential relations. We computed non-linear terms using the centered pattern correspondence variable. For example, we computed the quadratic term of pattern correspondence by multiplying the centered pattern correspondence variable with itself. We tested exponential relations to examine whether acceptance/relationship satisfaction increases up to a level of pattern correspondence and then levels off after that level. Then, we compared fit statistics of models [e.g., Akaike information criterion (AIC), Bayesian information criterion (BIC)] to identify the best model. In cases when AIC and BIC scores contradicted, we chose the best model based on BIC score; because BIC values consider the sample size. We also checked the differences between BIC values to assess the strength of the superiority between the models (Raftery, 1995).

For the mean-level match, we conducted piecewise regression analysis (Edwards, 1994; Griffin et al., 1999) to estimate two different regressions below and above the match point (i.e., the point where perceived partner ratings score equals ideal standards score) in one model (cf. Lee et al., 2008; Frost and Forrester, 2013). That is, we regressed acceptance/relationship satisfaction on the negative differences (perceived partner ratings score < ideal standards score) and positive differences (perceived partner ratings score > ideal standards score) in the same model to investigate whether the effects of consistency are similar at each piece. We also estimated two separate intercepts for these two pieces. Thus, we were able to compute separate slopes, effect sizes and intercepts for each negative and positive difference piece and compare them with each other using piecewise regression analysis (Griffin et al., 1999).

We first estimated linear associations in both negative and positive differences pieces of mean-level match. Although we did not expect any non-linear associations below and above the match point, in subsequent models, we also investigated the significance of quadratic effects at each piece. We again compared fit statistics of models to identify the best model.

### Descriptive Statistics and Correlations

Descriptive statistics for, and correlations among, study variables are presented in **Table 1**. Importantly, both pattern correspondence and mean-level match were positively related to acceptance and relationship satisfaction. Although these two indicators of consistency showed somewhat similar associations with other variables, the correlation between them (*r* = 0.15) supported our theoretical argument that they represent different constructs.

We also investigated the distributions of the two consistency indicators. Frequencies of pattern correspondence showed that 91% of the 970 scores across all time points (see **Table 1**) were positive (i.e., positive correlation between perceived partner attributes and ideal standards) and 8% of the scores were negative. Only in 1% of the scores was there no association between perceived partner attributes and ideal standards (i.e., *r* = 0).

Frequencies of mean-level match showed that among 1,074 cases (see **Table 1**), 51% of the scores were below zero (i.e., perceived partner attributes < ideal standards), 41% of the scores were above zero (i.e., perceived partner attributes > ideal standards), and 8% of the scores were zero (i.e., perceived partner attributes = ideal standards). The range of the scores below zero, [-2.63, 0], was somewhat larger than the range of the scores above zero, [0, 1.63]. The standard deviations at each piece were 0.37 and 0.29, respectively.

We also examined whether the study variables differed across gender. Results (**Table 2**) showed that other than the pattern correspondence, none of the variables differed between men and women. In terms of pattern correspondence, women reported higher levels of consistency than men.

**Table 2:** Study variables across gender.

	Men		Women			
Variable	*M*	*SD*	*M*	*SD*	*t(df)*	*p*
(1) Ideal standards	5.71	0.56	5.76	0.50	-1.61 (*1073*)	0.11
(2) Perceived partner ratings	5.65	0.61	5.66	0.55	-0.33 (*1077*)	0.74
(3) Acceptance	4.18	0.45	4.21	0.43	-1.07 (*1076*)	0.29
(4) Relationship satisfaction	111.14	10.59	110.02	11.06	-1.69 (*1078*)	0.09
(5) Pattern correspondence	0.44	0.31	0.50	0.30	-3.25 (*968*)	0.001
(6) Mean-level match	-0.06	0.51	-0.10	0.48	1.36 (*1072*)	0.18

*Results show the statistics across all three waves of data.*

### Over-Time Standard APIM Results for Acceptance

#### Pattern Correspondence

Using the over-time standard APIM, we regressed acceptance on both actor’s and partner’s pattern correspondence. We first examined whether there is a linear association between pattern correspondence and acceptance. In the linear model (see **Table 4**), neither actor’s nor partner’s pattern correspondence had an association with acceptance.

Comparison of the models presented in **Table 3** showed that the model with exponential relation between pattern correspondence and acceptance had the lowest BIC level. The detailed results of that model are presented in **Table 4**. Neither actor effect nor partner effect in the exponential model was significant.

**Table 3:** Estimates and fit statistics in the linear and non-linear models for acceptance.

Model	-2 Log Likelihood	# Parameters	Δ Chi Square	*p*	AIC	BIC
**Pattern correspondence models**						
Linear	700.06	8.00			716.06	754.26
Quadratic	695.47	10.00	4.59	0.10	715.47	763.22
Cubic	690.18	12.00	9.88	0.04	714.18	771.48
Negative exponential	698.46	8.00	-	-	714.46	752.66
**Mean-level match models**						
Linear	816.83	11.00			838.83	893.48
Quadratic	814.29	15.00	2.53	0.64	844.29	918.81

*AIC, Akaike information criterion; BIC, Bayesian information criterion. Δ Chi Square results represent the results of the comparison with the linear model. Quadratic and cubic models included lower level effects (e.g., linear effect in the quadratic model).*

**Table 4:** Estimates in the over-time standard APIM for pattern correspondence’s effect on acceptance.

						95% CI		
	Estimate	*SE*	*df*	*t*	*p*	Lower bound	Upper bound	*r*
**Linear model**								
Intercept	4.21	0.02	175.11	194.58	0.00	4.16	4.25	–
Actor consistency (Linear)	0.07	0.04	785.80	1.58	0.12	-0.02	0.15	0.06
Partner consistency (Linear)	0.05	0.04	789.13	1.19	0.24	-0.03	0.13	0.04
**Exponential model**								
Intercept	4.34	0.06	526.16	70.52	0.00	4.22	4.46	–
Actor consistency (negative exponential)	-0.07	0.04	780.60	-1.82	0.07	-0.14	0.01	0.07
Partner consistency (negative exponential)	-0.06	0.04	782.79	-1.55	0.12	-0.13	0.02	0.06

*Effect sizes are shown in the last column. For the results of models with higher BIC values in **Table 3**, please see Supplementary Materials.*

Although the model with smaller BIC is considered as the best-fitting model in the literature (Singer and Willett, 2003), Raftery (1995) argued that a BIC difference greater than 10 shows a strong support for the model with smaller BIC. As presented in **Table 3**, the BIC difference between the linear and exponential models was very little (<2, weak evidence, Raftery, 1995). Thus, we concluded that both linear and exponential models had similar fit levels when explaining the association between pattern correspondence and acceptance. As explained above, in the linear model too, neither actor effect nor partner effect was significant.

#### Mean-Level Match

**FIGURE 1:**
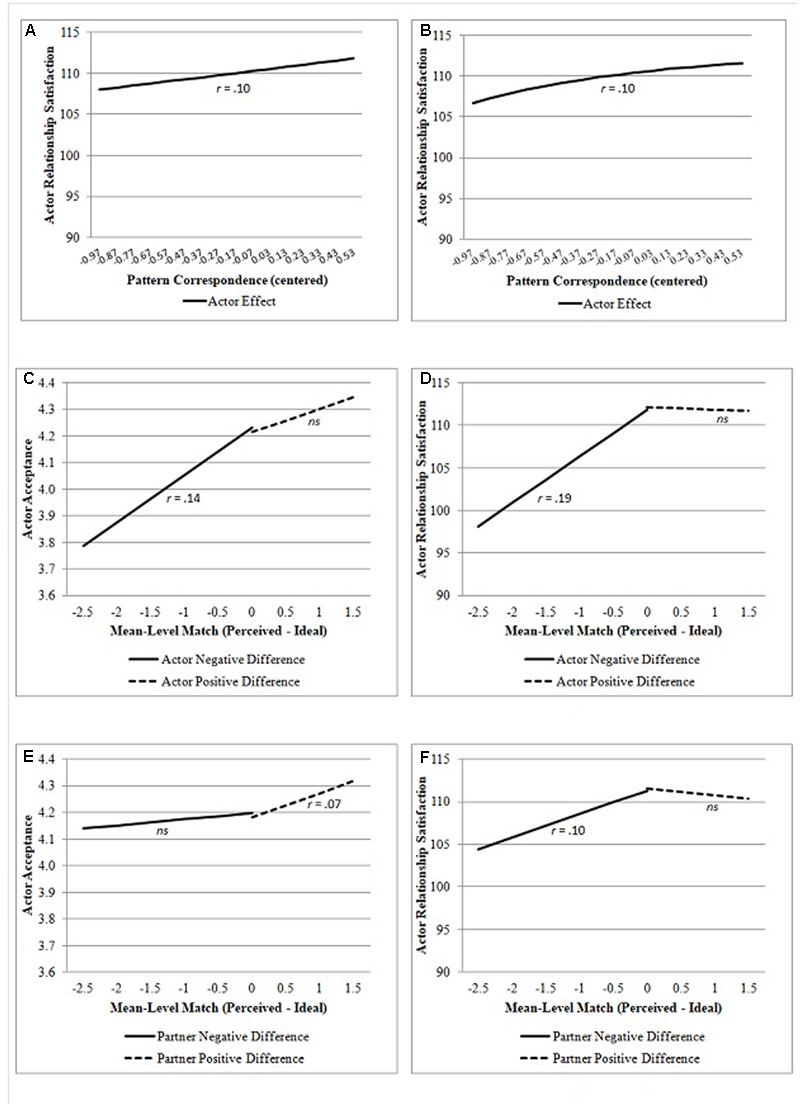
Associations of pattern correspondence **(A,B)** and mean-level match **(C–F)** with acceptance and relationship satisfaction. Figures include the effect sizes for the significant associations.

Using the over-time standard APIM for mean-level match, we regressed acceptance on both actor’s and partner’s negative differences and positive differences between perceived partner ratings and ideal standards. Results showed that the model with linear effects fit the data better than the model with quadratic effects (**Table 3**, i.e., lower AIC and BIC levels, BIC difference > 10). We present the results of the model with linear effects in **Table 5** and its graphs in **Figures 1C,E**.

**Table 5:** Estimates in the over-time standard APIM for mean-level match’s effect on acceptance.

						95% CI	
	Estimate	*SE*	*df*	*t*	*p*	Lower bound	Upper bound	*r*
Intercept for negative difference	4.22	0.03	480.26	151.62	0.00	4.17	4.28	-
Intercept for positive difference	4.21	0.03	675.47	131.05	0.00	4.14	4.27	-
Actor negative difference	0.18	0.04	987.90	4.47	0.00	0.10	0.26	0.14
Actor positive difference	0.09	0.05	854.95	1.60	0.11	-0.02	0.19	0.05
Partner negative difference	0.02	0.04	997.37	0.63	0.53	-0.05	0.10	0.02
Partner positive difference	0.09	0.04	881.91	2.10	0.04	0.01	0.18	0.07

*Effect sizes are shown in the last column. For the results of models with higher BIC values in **Table 3**, please see Supplementary Materials.*

The results indicated a significant positive association between actor’s mean-level match and acceptance up to the point where perceived partner rating score equals ideal standards score (**Figure 1C**). Having a perceived partner rating score higher than the ideal standards score was not significantly related to actor’s acceptance. As can be seen in **Figure 1E**, partner’s negative difference (i.e., partner’s perceived partner rating lower than his/her own ideal standards) was not related to actor’s acceptance. Nevertheless, there was a significant positive association between positive difference (i.e., partner’s perceived partner rating higher than his/her own ideal standards) and actor’s acceptance of the partner.

### Over-Time Standard APIM Results for Relationship Satisfaction

#### Pattern Correspondence

We again first examined a linear association between pattern correspondence and relationship satisfaction. The results in our APIM showed that although actor’s pattern correspondence had a linear association with relationship satisfaction, partner’s pattern correspondence did not have a significant linear association with relationship satisfaction (see **Table 7** and **Figure 1A**).

Comparison of the models in **Table 6** showed that an exponential relation between pattern correspondence and relationship satisfaction had the lowest levels of AIC and BIC. The detailed results of this model and its graph are presented in **Table 7** and **Figure 1B**, respectively. Actor’s consistency had an exponential association with his/her relationship satisfaction, but partner’s consistency did not.

**Table 6:** Estimates and fit statistics in the linear and non-linear models for relationship satisfaction.

Model	-2 Log Likelihood	# Parameters	Δ Chi Square	*p*	AIC	BIC
**Pattern correspondence models**						
Linear	6240.57	8.00			6256.57	6294.79
Quadratic	6238.23	10.00	2.34	0.31	6258.23	6306.01
Cubic	6237.14	12.00	3.43	0.49	6261.14	6318.47
Negative exponential	6239.69	8.00	-	-	6255.69	6293.91
**Mean-level match models**						
Linear	7482.33	11.00			7504.33	7558.99
Quadratic	7477.44	15.00	4.88	0.30	7507.44	7581.99

*AIC, Akaike information criterion; BIC, Bayesian information criterion. Δ Chi Square results represent the results of the comparison with the linear model. Quadratic and cubic models included lower level effects (e.g., linear effect in the quadratic model).*

**Table 7:** Estimates in the over-time standard APIM for the effect of pattern correspondence on relationship satisfaction.

						95% CI	
	Estimate	*SE*	*df*	*t*	*p*	Lower bound	Upper bound	*r*
**Linear model**								
Intercept	110.46	0.62	171.89	179.17	0.00	109.24	111.68	-
Actor consistency (linear)	2.54	0.98	722.25	2.60	0.01	0.62	4.46	0.10
Partner consistency (linear)	0.76	0.98	748.86	0.78	0.44	-1.16	2.68	0.03
**Exponential model**								
Intercept	113.50	1.49	465.98	76.08	0.00	110.57	116.43	-
Actor consistency (negative exponential)	-2.38	0.86	722.68	-2.78	0.01	-4.06	-0.70	0.10
Partner consistency (negative exponential)	-0.52	0.86	739.65	-0.61	0.54	-2.20	1.15	0.02

*Effect sizes are shown in the last column. For the results of models with higher BIC values in **Table 6**, please see Supplementary Materials.*

Again, the difference between BIC levels (<2, Raftery, 1995) indicated that linear and exponential models had similar fit levels (**Table 6**). Indeed, as reported above, the results for linear and exponential models were consistent. In both models, only actor effect was significant.

#### Mean-Level Match

The results in **Table 6** showed that the model with linear associations better represented the association between mean-level match and relationship satisfaction than the model with quadratic associations (i.e., lower AIC and BIC levels, BIC difference > 10). The results are given in **Table 8** and the graphs are presented in **Figures 1D,F**.

**Table 8:** Estimates in the over-time standard APIM for the effect of mean-level match on relationship satisfaction.

						95% CI	
	Estimate	*SE*	*df*	*t*	*p*	Lower bound	Upper bound	*r*
Intercept for negative difference	112.53	0.69	417.92	163.48	0.00	111.17	113.88	-
Intercept for positive difference	112.83	0.78	588.34	145.37	0.00	111.30	114.35	-
Actor negative difference	5.46	0.90	965.72	6.06	0.00	3.69	7.23	0.19
Actor positive difference	-0.25	1.22	833.66	-0.20	0.84	-2.65	2.15	0.01
Partner negative difference	2.73	0.83	972.95	3.29	0.00	1.10	4.37	0.10
Partner positive difference	-0.85	0.98	861.15	-0.87	0.39	-2.77	1.07	0.03

*Effect sizes are shown in the last column. For the results of models with higher BIC values in **Table 6**, please see Supplementary Materials.*

The results indicated that actor’s mean-level match was positively related to relationship satisfaction only when the perceived partner rating score was lower than the ideal standards score (**Figure 1D**). This finding was in line with the findings for acceptance. Different from the findings for acceptance, however, partner’s negative difference (i.e., partner’s perceived partner rating lower than his/her own ideal standards) was significantly related to actor’s relationship satisfaction (**Figure 1F**). There was no significant association at the positive difference piece. That is, the match between partner’s perception of actor’s attributes and partner’s ideals was positively associated with actor’s relationship satisfaction up to the point where partner’s perception of actor’s attributes equaled partner’s ideals.

### Over-Time Standard APIM Results for Each Dimension

Because we proposed that individuals will be less tolerable to larger discrepancies on the warmth dimension compared to the other two dimensions (vitality and status), we also examined our hypothesis for each dimension of the ISM separately and reported the results in our Supplementary Materials. In the analyses using the pattern correspondence, only one of the possible six actor effects (2 dependent variables^∗^3 dimensions) was significant. That is, actor’s consistency in the status dimension had a significant linear/exponential link with relationship satisfaction (i.e., linear and exponential models had similar levels of BIC). Contrary to our prediction, actor’s consistency in the warmth dimension was not significantly associated with acceptance or relationship satisfaction in our analyses using the pattern correspondence.

In the analyses for partner acceptance using the mean-level match, comparison of effect sizes across dimensions showed that the effect size in the warmth dimension was only slightly higher than the effect sizes in the other two dimensions (*r* = 0.10 vs. *r* = 0.09 and *r* = 0.07, Supplementary File, p. 10). In the analyses for relationship satisfaction, effect size in the warmth dimension was lower than the effect size in the vitality dimension (*r* = 0.17 vs. *r* = 0.22, Supplementary File, p. 11). Thus, results did not provide a strong evidence for our hypothesis that individuals are less tolerable to discrepancies in the warmth dimension than they are to discrepancies in the other two dimensions.

### Summary

Overall, analyses using pattern correspondence suggested that the association of within-person correlation between perceived partner ratings and ideal standards with relationship satisfaction is either linear or exponential. We did not find a significant association between pattern correspondence and acceptance. The results on mean-level match suggested that both acceptance and relationship satisfaction predictably differ when actor’s perceived partner attributes fall short of ideal standards. Although partner’s negative difference and partner’s positive difference were predictive in some cases, those effects were not consistent across the two outcomes and their effect sizes were lower than the effect size of actor’s negative difference.

### Additional Analyses

#### Gender

We investigated whether our results remained when we controlled for gender. Gender did not reach significance in any of the models, and did not affect the results we reported above. We also checked whether our results varied across men and women, and there was no significant interaction between our results and gender, which indicated that our findings hold for both genders (see Supplementary Materials for detailed results).

#### Acceptance and Consistency Interaction

One can also argue that acceptance buffers the effect of discrepancy between perceived partner ratings and ideal standards on relationship satisfaction (Baucom et al., 1996; Karremans et al., 2017). That is, even when there is a gap between one’s ideals and partner attributes, one’s relationship satisfaction may not be affected if one accepts the partner (see also Campbell et al., 2001). Thus, we investigated the interactive effect of consistency with acceptance on relationship satisfaction. We used the models we reported above for relationship satisfaction as base models (**Tables 7, 8**) and added the main effect of acceptance and interaction terms to those models. For example, we added acceptance, interaction between actor consistency and acceptance, and interaction between partner consistency and acceptance to the base model using pattern correspondence in **Table 7**.

Results revealed some significant interactions both using pattern correspondence and mean-level match. For pattern correspondence, actor’s consistency was related to actor’s own relationship satisfaction depending on the acceptance level in the linear model, *b* = -4.44, *t*(784.38) = -2.14, 95% CI = [-8.52, -0.36], *p* = 0.03, *r* = 0.08. In the exponential model, this effect was not significant, *b* = 3.52, *t*(777.70) = 1.94, 95% CI = [-0.03, 0.07], *p* = 0.05, *r* = 0.07. Simple slope analyses showed that consistency was related to relationship satisfaction only among individuals who had low levels of acceptance (i.e., one standard deviation below the mean) in the linear model, *b* = 4.33, *t*(781.90) = 3.34, 95% CI = [1.79, -6.88], *p* = 0.001, *r* = 0.12. This association was not significant among individuals who had high levels of acceptance (i.e., one standard deviation above the mean), *b* = 0.44, *t*(806.97) = 0.33, 95% CI = [-2.19, 3.08], *p* = 0.74, *r* = 0.01. These results suggested that discrepancy between partner attributes and ideal standards was not destructive for relationship satisfaction when an actor accepts the partner.

The model using mean-level match showed that acceptance interacted with actor’s negative difference, *b* = -3.15, *t*(948.24) = -2.10, 95% CI = [-6.09, -0.21], *p* = 0.04, *r* = 0.07, but not with actor’s positive difference, partner’s negative difference, or partner’s positive difference. Thus, the results reported for actor’s positive difference, partner’s negative difference, or partner’s positive difference in **Table 8** was constant across different levels of actor’s acceptance. Simple slope analyses showed that actor negative difference (i.e., actor’s perceived partner attributes lower than actor’s ideal standards) was more influential on relationship satisfaction among individuals with low levels of acceptance, *b* = 5.53, *t*(993.76) = 5.70, 95% CI = [3.63, 7.43], *p* < 0.001, *r* = 0.18, than among individuals with high levels of acceptance, *b* = 2.77, *t*(931.91) = 2.20, 95% CI = [0.29, 5.24], *p* = 0.03, *r* = 0.07. These results suggested that individuals who accepted their partner were less affected by the discrepancies between their ideal standards and perceived partner ratings than individuals who did not accept their partner.

#### Ideal Standards and Perceived Partner Ratings

We also investigated if our results remained controlling for the effects of ideal standards and perceived partner ratings. We were able to conduct this analysis for pattern correspondence, but not for mean-level match because of its computation method. That is, it used a signed difference between two scores, meaning that it is not possible to include both elements of the difference in the analysis. Furthermore, controlling for only one element of the difference (e.g., ideal standards) results in the difference variable representing the conditional main effect of the non-included element of the difference (e.g., perceived partner ratings), not the difference between the elements (Edwards, 1994).

When ideal standards and perceived partner ratings were added to the models with pattern correspondence in **Table 4**, again, neither actor effect nor partner effect was significant (Actor effect: *b* = 0.07, *t*(827.91) = 1.86, 95% CI = [0.00, 0.15], *p* = 0.06, *r* = 0.06 in the linear model and *b* = -0.06, *t*(823.80) = -1.98, 95% CI = [-0.14, 0.00], *p* = 0.05, *r* = 0.07 in the exponential model; Partner effect: *b* = 0.06, *t*(833.31) = 1.41, 95% CI = [-0.02, 0.13], *p* = 0.16, *r* = 0.05 in the linear model and *b* = -0.06, *t*(816.31) = -1.71, 95% CI = [-0.13, 0.01], *p* = 0.09, *r* = 0.06 in the exponential model). When we re-ran the model presented in **Table 7** controlling for ideal standards and perceived partner ratings, results stayed almost the same. That is, actor effect on relationship satisfaction was significant whereas partner effect was not (Actor effect: *b* = 2.83, *t*(773.58) = 3.05, 95% CI = [1.01, 4.65], *p* = 0.002, *r* = 0.11 in the linear model and *b* = -2.52, *t*(773.58) = -3.11, 95% CI = [-4.11, -0.93], *p* = 0.002, *r* = 0.11 in the exponential model; Partner effect: *b* = 1.02, *t*(806.29) = 1.11, 95% CI = [-0.79, 2.84], *p* = 0.27, *r* = 0.04 in the linear model and *b* = -0.66, *t*(793.06) = -0.82, 95% CI = [-2.25, 0.93], *p* = 0.41, *r* = 0.03 in the exponential model).

## Discussion

In this dyadic study with repeated assessments over time, we found initial evidence that consistency between perceived partner attributes and ideal standards seems to have a non-linear association with both acceptance and relationship satisfaction (albeit the results vary across consistency indicators, see below). Discrepancies between perceived partner attributes and ideal standards were related to partner acceptance and relationship satisfaction, especially when the perceived partner attributes were short of ideal standards. When perceived partner attributes exceeded ideal standards, discrepancies were not related to acceptance or relationship satisfaction in almost any of our examinations. Our results thus indicated that previous findings on the linear association between the discrepancies and relationship satisfaction may be accounted for by the lower satisfaction in the presence of negative difference rather than higher satisfaction in the presence of positive difference.

There were, however, some differences in the findings across the two consistency indicators (i.e., pattern correspondence and mean-level match), across two dependent variables (i.e., partner acceptance and relationship satisfaction), and across actor and partner effects. First, effect sizes showed that mean-level match had a stronger relation than pattern correspondence with relationship satisfaction. This result indicates that individuals value the average match between their partner’s attributes and their own ideals more than they value the consistency between the patterns of partner attributes and ideal standards. Nevertheless, this result should be interpreted cautiously until replicated, because most of our participants had positive correlations between the patterns of partner attributes and ideal standards, which limited the variance in pattern correspondence. Second, consistency parameters had either no association with partner acceptance, or a weaker association than with relationship satisfaction. Perhaps partner acceptance is related to individual differences such as mindfulness and thus less likely than relationship satisfaction to be affected by the consistency (Karremans et al., 2017). Or, perhaps because newlywed individuals are highly committed to their relationships, they may accept low levels of consistency even if they are not satisfied (Rusbult et al., 1998). Examinations of these possible explanations await future research. Third, most actor effects were significant whereas most partner effects were not, and significant partner effects had smaller effect sizes than significant actor effects. This finding is in line with the previous dyadic and family research showing that actor effects are generally stronger than partner effects (Kenny et al., 2001; Eichelsheim et al., 2009).

Our findings did not support the hypothesis that consistency in the warmth dimension has a stronger association with acceptance and relationship satisfaction than consistencies in the vitality and status dimensions. Nevertheless, a more thorough comparison across the ISM dimensions necessitates having all the dimensions and predictors in the same statistical model, which we could not do because of the power concerns and for preventing our analyses to become more complex. Therefore, this result should be interpreted cautiously until replicated.

In accordance with previous research (Campbell et al., 2001), this study also showed that being more accepting of a partner minimized the association between perceived discrepancies and relationship satisfaction. Future studies should examine when and how individuals accept, rather than have positive illusions about or try to regulate, their partner in case of discrepancies between perceived partner attributes and ideal standards (Murray et al., 1996; Overall et al., 2006; Karremans et al., 2017). Perhaps partners try to regulate their partner’s behaviors when the discrepancies are large, but have positive illusions when the discrepancies are only minor.

Given that, in the present study, partner acceptance protected people’s relationship satisfaction from the negative impact of perceived discrepancies, future studies should further examine partner acceptance and its potential benefits. Is partner acceptance an individual difference or does it occur only in certain relationships (e.g., relationships with high levels of closeness), and can it be learned or trained (Carson et al., 2007; Karremans et al., 2017)? Although contemporary couple intervention protocols include techniques to foster acceptance (e.g., Jacobson et al., 2000), research on partner acceptance is still limited. Other promising future directions include, but are not limited to, longitudinal investigations of changes in ideal standards and perceived partner attributes, and the effect of one’s perception of his/her own attributes on partner acceptance and relationship satisfaction.

## Conclusion

This research showed that individuals are more sensitive to negative discrepancies (i.e., perceived partner attributes < ideal standards) than they are to positive discrepancies (i.e., perceived partner attributes > ideal standards) in the comparison between perceived partner ratings and ideal standards in marriages. Although any negative discrepancy had a negative association with partner acceptance/relationship satisfaction, positive discrepancies did not add much to the prediction of partner acceptance/relationship satisfaction. Considering our additional finding that the effect of negative discrepancies diminished significantly among accepting participants, future studies should investigate whether and how acceptance can be fostered in individuals who perceive that their partner’s attributes fall short of their ideals.

## Author Contributions

CF designed the study and collected the data. AB-T, LC, and CF developed the initial research question, JK and GK contributed to the improvement of the initial idea. AB-T conducted the analysis and wrote the first draft of the paper. AB-T, LC, CF, JK, and GK worked on the first draft and revised the conceptualizations, operationalizations, and manuscript altogether. All authors approved the final version of the manuscript.

## Conflict of Interest Statement

The authors declare that the research was conducted in the absence of any commercial or financial relationships that could be construed as a potential conflict of interest.

## Footnotes

1 Existing research sometimes refers to a discrepancy between ideal standards and perceived partner ratings, which ranges from “no discrepancy” to “complete discrepancy” (e.g., Campbell et al., 2001). Lower and higher levels on that continuum refer to higher and lower levels of consistency between ideal standards and perceived partner attributes, respectively (e.g., Fletcher et al., 2000). Thus, in this paper greater consistency refers to lower discrepancy, and vice versa.

2 Due to the lack of space, consistency between ideal standards and perceived partner ratings is referred to as *consistency* throughout the text.

3 Hypotheses of this study were pre-registered on the Open Science Framework (https://osf.io/fqbcy/?view_only=74fea2c276ab49919d036e986256a751).

4 A discussion of the methods was also placed on the Open Science Framework (https://osf.io/mc8zy/?view_only=6c5a311169c44554b0cfe06fdefb259a).
