# Supplementary material for: Ideal Standards, Acceptance, and Relationship Satisfaction: Latitudes of Differential Effects
Source: Front Psychol. 2017 Sep 28;8:1691. doi: 10.3389/fpsyg.2017.01691 (PMC5625328; doi:10.3389/fpsyg.2017.01691)
Supplement: Supplementary file 1 [file Table_1.docx]

**SUPPLEMENTARY FILE**

**Models with Higher BIC Values**

Results of models with higher BIC values in Table 3 and Table 6 in our paper are presented in the following pages (pp. 2-5).

**Dimensions of the Ideal Standards Model**

To conduct our analyses for each dimension of the Ideal Standards Model, namely warmth/trustworthiness, vitality/passion, and status/resources, we first examined the dimensions of the scale in our sample. We conducted exploratory factor analysis with three factors using all 16 items (understanding, supportive, kind, good listener, sensitive, trustworthy, sexy, pays attention to his/her appearance, attractive appearance, good lover, adventurous, original, creative, inventive, successful, ambitious). We examined the contents of each dimension and item, loadings of items, and cross-loadings. Based on these examinations, we could not add two items (sensitive and original) to any of the dimensions. The remaining items however, showed the pattern in the Ideal Standards Model. Three dimensions and the items loaded on those items were as follows: warmth/trustworthiness (understanding, supportive, kind, good listener, trustworthy), vitality/passion (sexy, pays attention to his/her appearance, attractive appearance, good lover), and status/resources (adventurous, creative, inventive, successful, ambitious). This three-dimension structure had acceptable fit statistics using both ideal standards (*χ*^2^ (74, N = 1075) = 620.16 (*p* < .05), Comparative Fit Index (CFI) = .91, and Root Mean Square Error of Approximation (RMSEA) of .08 [90% CI = .08, .09]) and perceived partner ratings (*χ*^2^ (74, N = 1079) = 780.41 (*p* < .05), CFI = .89, and RMSEA of .09 [90% CI = .09, .10]). The fit statistics of the linear and nonlinear models with each dimension, and the estimates for each dimension are reported in the following pages (pp. 6-11).

**Gender**

Results of the analyses controlling for gender and examining gender interactions are also presented in the following pages (pp. 12-19).

| *Estimates in the Over-Time Standard APIM for Pattern Correspondence's Effect on Acceptance* | | | | | | | | |
| --- | --- | --- | --- | --- | --- | --- | --- | --- |
|  |  |  |  |  |  | 95% CI | |  |
|  | Estimate | *SE* | *df* | *t* | *p* | LB | UB | *r* |
| Quadratic Model |  |  |  |  |  |  |  |  |
| Intercept | 4.24 | 0.03 | 305.80 | 161.69 | 0.00 | 4.19 | 4.29 | - |
| Actor Consistency (Linear) | 0.04 | 0.05 | 779.81 | 0.90 | 0.37 | -0.05 | 0.13 | 0.03 |
| Partner Consistency (Linear) | 0.02 | 0.05 | 783.73 | 0.44 | 0.66 | -0.07 | 0.11 | 0.02 |
| Actor Consistency (Quadratic) | -0.16 | 0.11 | 711.37 | -1.47 | 0.14 | -0.38 | 0.06 | 0.06 |
| Partner Consistency (Quadratic) | -0.19 | 0.11 | 709.48 | -1.69 | 0.09 | -0.41 | 0.03 | 0.06 |
| Cubic Model |  |  |  |  |  |  |  |  |
| Intercept | 4.23 | 0.03 | 345.81 | 154.51 | 0.00 | 4.18 | 4.29 | - |
| Actor Consistency (Linear) | 0.08 | 0.07 | 718.61 | 1.13 | 0.26 | -0.06 | 0.21 | 0.04 |
| Partner Consistency (Linear) | -0.09 | 0.07 | 723.32 | -1.30 | 0.19 | -0.22 | 0.05 | 0.05 |
| Actor Consistency (Quadratic) | -0.22 | 0.15 | 663.31 | -1.49 | 0.14 | -0.51 | 0.07 | 0.06 |
| Partner Consistency (Quadratic) | 0.01 | 0.15 | 660.71 | 0.07 | 0.95 | -0.28 | 0.30 | 0.00 |
| Actor Consistency (Cubic) | -0.20 | 0.28 | 639.83 | -0.69 | 0.49 | -0.76 | 0.36 | 0.03 |
| Partner Consistency (Cubic) | 0.59 | 0.28 | 642.11 | 2.09 | 0.04 | 0.04 | 1.15 | 0.08 |
| *Note.* LB = Lower bound. UB = Upper bound. | | | | | | | |  |

| *Estimates in the Over-Time Standard APIM for Mean-Level Match's Effect on Acceptance* | | | | | | | |  |
| --- | --- | --- | --- | --- | --- | --- | --- | --- |
|  |  |  |  |  |  | 95% CI | | |
|  | Estimate | *SE* | *df* | *t* | *p* | LB | UB | *r* |
| Quadratic Model |  |  |  |  |  |  |  |  |
| Intercept for negative difference | 4.23 | 0.03 | 622.59 | 130.03 | 0.00 | 4.17 | 4.29 | - |
| Intercept for positive difference | 4.19 | 0.04 | 858.54 | 102.35 | 0.00 | 4.11 | 4.27 | - |
| Actor negative difference (Linear) | 0.17 | 0.08 | 868.45 | 2.05 | 0.04 | 0.01 | 0.33 | 0.07 |
| Actor positive difference (Linear) | 0.23 | 0.15 | 833.31 | 1.52 | 0.13 | -0.07 | 0.54 | 0.05 |
| Partner negative difference (Linear) | 0.10 | 0.08 | 879.34 | 1.33 | 0.18 | -0.05 | 0.25 | 0.04 |
| Partner positive difference (Linear) | 0.08 | 0.11 | 849.72 | 0.73 | 0.46 | -0.13 | 0.29 | 0.03 |
| Actor negative difference (Quadratic) | -0.01 | 0.05 | 831.81 | -0.12 | 0.90 | -0.11 | 0.10 | 0.00 |
| Actor positive difference (Quadratic) | -0.13 | 0.13 | 840.41 | -1.03 | 0.30 | -0.38 | 0.12 | 0.04 |
| Partner negative difference (Quadratic) | 0.06 | 0.05 | 845.24 | 1.21 | 0.23 | -0.04 | 0.16 | 0.04 |
| Partner positive difference (Quadratic) | 0.00 | 0.10 | 854.55 | -0.03 | 0.98 | -0.20 | 0.20 | 0.00 |
| *Note.* LB = Lower bound. UB = Upper bound. | | | | | | | |  |

| *Estimates in the Over-Time Standard APIM for Pattern Correspondence's Effect on Relationship Satisfaction* | | | | | | | |  |
| --- | --- | --- | --- | --- | --- | --- | --- | --- |
|  |  |  |  |  |  | 95% CI | | |
|  | Estimate | *SE* | *df* | *t* | *p* | LB | UB | *r* |
| Quadratic Model |  |  |  |  |  |  |  |  |
| Intercept | 110.53 | 0.71 | 270.70 | 156.74 | 0.00 | 109.14 | 111.92 | - |
| Actor Consistency (Linear) | 2.08 | 1.05 | 715.18 | 1.97 | 0.05 | 0.01 | 4.15 | 0.07 |
| Partner Consistency (Linear) | 1.09 | 1.05 | 746.02 | 1.03 | 0.30 | -0.98 | 3.16 | 0.04 |
| Actor Consistency (Quadratic) | -3.02 | 2.58 | 689.09 | -1.17 | 0.24 | -8.08 | 2.04 | 0.04 |
| Partner Consistency (Quadratic) | 2.25 | 2.57 | 666.11 | 0.87 | 0.38 | -2.81 | 7.30 | 0.03 |
| Cubic Model |  |  |  |  |  |  |  |  |
| Intercept | 110.34 | 0.73 | 300.17 | 151.78 | 0.00 | 108.91 | 111.77 | - |
| Actor Consistency (Linear) | 1.13 | 1.60 | 668.78 | 0.70 | 0.48 | -2.01 | 4.26 | 0.03 |
| Partner Consistency (Linear) | 0.19 | 1.60 | 696.61 | 0.12 | 0.90 | -2.94 | 3.32 | 0.00 |
| Actor Consistency (Quadratic) | -1.14 | 3.42 | 632.06 | -0.33 | 0.74 | -7.85 | 5.58 | 0.01 |
| Partner Consistency (Quadratic) | 4.04 | 3.42 | 632.68 | 1.18 | 0.24 | -2.68 | 10.76 | 0.05 |
| Actor Consistency (Cubic) | 5.30 | 6.57 | 617.70 | 0.81 | 0.42 | -7.60 | 18.19 | 0.03 |
| Partner Consistency (Cubic) | 5.01 | 6.56 | 621.81 | 0.76 | 0.45 | -7.87 | 17.89 | 0.03 |
| *Note.* LB = Lower bound. UB = Upper bound. | | | | | | | |  |

| *Estimates in the Over-Time Standard APIM for Mean-Level Match's Effect on Relationship Satisfaction* | | | | | | | |  |
| --- | --- | --- | --- | --- | --- | --- | --- | --- |
|  |  |  |  |  |  | 95% CI | | |
|  | Estimate | *SE* | *df* | *t* | *p* | LB | UB | *r* |
| Quadratic Model |  |  |  |  |  |  |  |  |
| Intercept for negative difference | 113.14 | 0.78 | 558.23 | 144.15 | 0.00 | 111.60 | 114.68 | - |
| Intercept for positive difference | 112.63 | 0.96 | 811.80 | 116.83 | 0.00 | 110.73 | 114.52 | - |
| Actor negative difference (Linear) | 8.82 | 1.86 | 845.72 | 4.74 | 0.00 | 5.17 | 12.47 | 0.16 |
| Actor positive difference (Linear) | 1.32 | 3.48 | 816.18 | 0.38 | 0.71 | -5.51 | 8.14 | 0.01 |
| Partner negative difference (Linear) | 2.58 | 1.72 | 854.74 | 1.50 | 0.13 | -0.79 | 5.96 | 0.05 |
| Partner positive difference (Linear) | -1.78 | 2.43 | 832.77 | -0.73 | 0.46 | -6.55 | 2.99 | 0.03 |
| Actor negative difference (Quadratic) | 2.43 | 1.18 | 808.81 | 2.06 | 0.04 | 0.11 | 4.74 | 0.07 |
| Actor positive difference (Quadratic) | -1.35 | 2.89 | 824.13 | -0.47 | 0.64 | -7.03 | 4.32 | 0.02 |
| Partner negative difference (Quadratic) | -0.23 | 1.13 | 820.39 | -0.21 | 0.84 | -2.45 | 1.98 | 0.01 |
| Partner positive difference (Quadratic) | 1.06 | 2.32 | 832.20 | 0.46 | 0.65 | -3.50 | 5.62 | 0.02 |
| *Note.* LB = Lower bound. UB = Upper bound. | | | | | | | |  |

|  |  |  |  |  |  |  |
| --- | --- | --- | --- | --- | --- | --- |
| Estimates and Fit Statistics in the Linear and Nonlinear Models for Acceptance Using the Warmth Dimension | | | | | | |
| Model | -2 Log Likelihood | # parameters | ∆ Chi Square | *p* | AIC | BIC |
| *Pattern Correspondence Models* | |  |  |  |  |  |
| Linear | 168.78 | 8.00 |  |  | 184.78 | 210.59 |
| Quadratic | 165.84 | 10.00 | 2.94 | 0.23 | 185.84 | 218.10 |
| Cubic | 164.29 | 12.00 | 4.49 | 0.34 | 188.29 | 227.00 |
| Negative Exponential | 170.37 | 8.00 | - | - | 186.37 | 212.18 |
| *Mean-Level Match Models* |  |  |  |  |  |  |
| Linear | 830.81 | 11.00 |  |  | 852.81 | 907.46 |
| Quadratic | 817.94 | 15.00 | 12.87 | 0.01 | 847.94 | 922.46 |
|  |  |  |  |  |  |  |
| Estimates and Fit Statistics in the Linear and Nonlinear Models for Acceptance Using the Vitality Dimension | | | | | | |
| Model | -2 Log Likelihood | # parameters | ∆ Chi Square | *p* | AIC | BIC |
| *Pattern Correspondence Models* | |  |  |  |  |  |
| Linear | 278.83 | 8.00 |  |  | 294.83 | 324.62 |
| Quadratic | 277.33 | 10.00 | 1.49 | 0.47 | 297.33 | 334.57 |
| Cubic | 276.87 | 12.00 | 1.96 | 0.74 | 300.87 | 345.55 |
| Negative Exponential | 279.77 | 8.00 | - | - | 295.77 | 325.56 |
| *Mean-Level Match Models* |  |  |  |  |  |  |
| Linear | 834.33 | 11.00 |  |  | 856.33 | 910.97 |
| Quadratic | 831.08 | 15.00 | 3.24 | 0.52 | 861.08 | 935.60 |
|  |  |  |  |  |  |  |
| Estimates and Fit Statistics in the Linear and Nonlinear Models for Acceptance Using the Status Dimension | | | | | | |
| Model | -2 Log Likelihood | # parameters | ∆ Chi Square | *p* | AIC | BIC |
| *Pattern Correspondence Models* | |  |  |  |  |  |
| Linear | 556.05 | 8.00 |  |  | 572.05 | 608.44 |
| Quadratic | 555.32 | 10.00 | 0.74 | 0.69 | 575.32 | 620.80 |
| Cubic | 554.45 | 12.00 | 1.60 | 0.81 | 578.45 | 633.03 |
| Negative Exponential | 555.93 | 8.00 | - | - | 571.93 | 608.32 |
| *Mean-Level Match Models* |  |  |  |  |  |  |
| Linear | 839.35 | 11.00 |  |  | 861.35 | 916.00 |
| Quadratic | 834.64 | 15.00 | 4.71 | 0.32 | 864.64 | 939.16 |

| Estimates and Fit Statistics in the Linear and Nonlinear Models for Relationship Satisfaction Using the Warmth Dimension | | | | | | |
| --- | --- | --- | --- | --- | --- | --- |
| Model | -2 Log Likelihood | # parameters | ∆ Chi Square | *p* | AIC | BIC |
| *Pattern Correspondence Models* | |  |  |  |  |  |
| Linear | 1338.16 | 8.00 |  |  | 1354.16 | 1379.96 |
| Quadratic | 1338.06 | 10.00 | 0.10 | 0.95 | 1358.06 | 1390.31 |
| Cubic | 1337.23 | 12.00 | 0.93 | 0.92 | 1361.23 | 1399.94 |
| Negative Exponential | 1338.17 | 8.00 | - | - | 1354.17 | 1379.97 |
| *Mean-Level Match Models* |  |  |  |  |  |  |
| Linear | 7508.25 | 11.00 |  |  | 7530.25 | 7584.91 |
| Quadratic | 7505.40 | 15.00 | 2.85 | 0.58 | 7535.40 | 7609.94 |
|  |  |  |  |  |  |  |
| Estimates and Fit Statistics in the Linear and Nonlinear Models for Relationship Satisfaction Using the Vitality Dimension | | | | | | |
| Model | -2 Log Likelihood | # parameters | ∆ Chi Square | *p* | AIC | BIC |
| *Pattern Correspondence Models* | |  |  |  |  |  |
| Linear | 2156.44 | 8.00 |  |  | 2172.44 | 2202.28 |
| Quadratic | 2154.99 | 10.00 | 1.45 | 0.48 | 2174.99 | 2212.29 |
| Cubic | 2152.80 | 12.00 | 3.65 | 0.46 | 2176.80 | 2221.56 |
| Negative Exponential | 2156.81 | 8.00 | - | - | 2172.81 | 2202.65 |
| *Mean-Level Match Models* |  |  |  |  |  |  |
| Linear | 7486.73 | 11.00 |  |  | 7508.73 | 7563.40 |
| Quadratic | 7479.24 | 15.00 | 7.49 | 0.11 | 7509.24 | 7583.78 |
|  |  |  |  |  |  |  |
| Estimates and Fit Statistics in the Linear and Nonlinear Models for Relationship Satisfaction Using the Status Dimension | | | | | | |
| Model | -2 Log Likelihood | # parameters | ∆ Chi Square | *p* | AIC | BIC |
| *Pattern Correspondence Models* | |  |  |  |  |  |
| Linear | 5053.92 | 8.00 |  |  | 5069.92 | 5106.33 |
| Quadratic | 5049.66 | 10.00 | 4.26 | 0.12 | 5069.66 | 5115.17 |
| Cubic | 5038.52 | 12.00 | 15.40 | 0.00 | 5062.52 | 5117.13 |
| Negative Exponential | 5052.02 | 8.00 | - | - | 5068.02 | 5104.43 |
| *Mean-Level Match Models* |  |  |  |  |  |  |
| Linear | 7517.41 | 11.00 |  |  | 7539.41 | 7594.08 |
| Quadratic | 7506.59 | 15.00 | 10.82 | 0.03 | 7536.59 | 7611.13 |

| *Estimates in the Over-Time Standard APIM for Pattern Correspondence's Effect on Acceptance* | | | | | | | |  |
| --- | --- | --- | --- | --- | --- | --- | --- | --- |
|  |  |  |  |  |  | 95% CI | | |
|  | Estimate | *SE* | *df* | *t* | *p* | Lower bound | Upper bound | *r* |
| Warmth |  |  |  |  |  |  |  |  |
| Intercept | 4.19 | 0.03 | 71.62 | 125.87 | 0.00 | 4.13 | 4.26 | - |
| Actor Consistency (Linear) | 0.00 | 0.05 | 150.00 | -0.05 | 0.96 | -0.11 | 0.10 | 0.00 |
| Partner Consistency (Linear) | -0.10 | 0.05 | 150.55 | -1.84 | 0.07 | -0.21 | 0.01 | 0.15 |
| Vitality |  |  |  |  |  |  |  |  |
| Intercept | 4.24 | 0.03 | 99.45 | 141.53 | 0.00 | 4.18 | 4.30 | - |
| Actor Consistency (Linear) | 0.04 | 0.04 | 234.97 | 1.13 | 0.26 | -0.03 | 0.11 | 0.07 |
| Partner Consistency (Linear) | 0.07 | 0.04 | 234.59 | 1.96 | 0.05 | 0.00 | 0.14 | 0.13 |
| Status |  |  |  |  |  |  |  |  |
| Intercept | 4.21 | 0.02 | 161.11 | 181.94 | 0.00 | 4.17 | 4.26 | - |
| Actor Consistency (Linear) | 0.04 | 0.03 | 552.58 | 1.48 | 0.14 | -0.01 | 0.09 | 0.06 |
| Partner Consistency (Linear) | 0.02 | 0.03 | 547.78 | 0.74 | 0.46 | -0.03 | 0.07 | 0.03 |
| Status |  |  |  |  |  |  |  |  |
| Intercept | 4.26 | 0.04 | 402.60 | 104.84 | 0.00 | 4.18 | 4.34 | - |
| Actor Consistency (Negative Exponential) | -0.03 | 0.02 | 538.41 | -1.45 | 0.15 | -0.06 | 0.01 | 0.06 |
| Partner Consistency (Negative Exponential) | -0.02 | 0.02 | 531.77 | -0.96 | 0.34 | -0.06 | 0.02 | 0.04 |

*Note.* Because BIC difference between linear and exponential models for status dimension was less than 2, we presented both models in the table.

| *Estimates in the Over-Time Standard APIM for Pattern Correspondence's Effect on Relationship Satisfaction* | | | | | | | |  |
| --- | --- | --- | --- | --- | --- | --- | --- | --- |
|  |  |  |  |  |  | 95% CI | | |
|  | Estimate | *SE* | *df* | *t* | *p* | Lower bound | Upper bound | *r* |
| Warmth |  |  |  |  |  |  |  |  |
| Intercept | 108.58 | 1.07 | 70.63 | 101.54 | 0.00 | 106.45 | 110.71 | - |
| Actor Consistency (Linear) | 0.55 | 1.19 | 81.81 | 0.46 | 0.65 | -1.83 | 2.92 | 0.05 |
| Partner Consistency (Linear) | -0.03 | 1.18 | 97.05 | -0.03 | 0.98 | -2.38 | 2.31 | 0.00 |
| Vitality |  |  |  |  |  |  |  |  |
| Intercept | 110.95 | 0.77 | 97.40 | 144.61 | 0.00 | 109.43 | 112.48 | - |
| Actor Consistency (Linear) | 1.29 | 0.75 | 201.66 | 1.72 | 0.09 | -0.19 | 2.76 | 0.12 |
| Partner Consistency (Linear) | 0.47 | 0.75 | 206.43 | 0.63 | 0.53 | -1.00 | 1.94 | 0.04 |
| Status |  |  |  |  |  |  |  |  |
| Intercept | 110.38 | 0.66 | 161.83 | 166.78 | 0.00 | 109.07 | 111.68 | - |
| Actor Consistency (Linear) | 1.89 | 0.64 | 555.58 | 2.95 | 0.00 | 0.63 | 3.15 | 0.12 |
| Partner Consistency (Linear) | -0.09 | 0.64 | 534.38 | -0.15 | 0.88 | -1.35 | 1.16 | 0.01 |
| Status |  |  |  |  |  |  |  |  |
| Intercept | 112.29 | 1.06 | 368.40 | 106.13 | 0.00 | 110.20 | 114.37 | - |
| Actor Consistency (Negative Exponential) | -1.56 | 0.47 | 534.35 | -3.29 | 0.00 | -2.49 | -0.63 | 0.14 |
| Partner Consistency (Negative Exponential) | -0.11 | 0.47 | 517.42 | -0.23 | 0.82 | -1.03 | 0.82 | 0.01 |

*Note.* Because BIC difference between linear and exponential models for status dimension was less than 2, we presented both models in the table.

| *Estimates in the Over-Time Standard APIM for Mean-Level Match's Effect on Acceptance* | | | | | | | |  |
| --- | --- | --- | --- | --- | --- | --- | --- | --- |
|  |  |  |  |  |  | 95% CI | | |
|  | Estimate | *SE* | *df* | *t* | *p* | Lower bound | Upper bound | *r* |
| Warmth |  |  |  |  |  |  |  |  |
| Intercept for negative difference | 4.20 | 0.03 | 392.81 | 161.78 | 0.00 | 4.15 | 4.25 | - |
| Intercept for positive difference | 4.24 | 0.03 | 770.53 | 122.14 | 0.00 | 4.17 | 4.31 | - |
| Actor negative difference | 0.11 | 0.03 | 976.34 | 3.29 | 0.00 | 0.05 | 0.18 | 0.10 |
| Actor positive difference | 0.01 | 0.04 | 859.07 | 0.13 | 0.90 | -0.08 | 0.09 | 0.00 |
| Partner negative difference | 0.03 | 0.03 | 991.47 | 0.79 | 0.43 | -0.04 | 0.09 | 0.03 |
| Partner positive difference | 0.07 | 0.03 | 897.70 | 2.05 | 0.04 | 0.00 | 0.13 | 0.07 |
| Vitality |  |  |  |  |  |  |  |  |
| Intercept for negative difference | 4.21 | 0.03 | 422.98 | 154.85 | 0.00 | 4.15 | 4.26 | - |
| Intercept for positive difference | 4.23 | 0.04 | 787.90 | 118.54 | 0.00 | 4.16 | 4.30 | - |
| Actor negative difference | 0.07 | 0.02 | 965.96 | 2.68 | 0.01 | 0.02 | 0.11 | 0.09 |
| Actor positive difference | 0.05 | 0.04 | 830.62 | 1.23 | 0.22 | -0.03 | 0.13 | 0.04 |
| Partner negative difference | 0.02 | 0.02 | 970.91 | 0.83 | 0.41 | -0.03 | 0.06 | 0.03 |
| Partner positive difference | 0.02 | 0.03 | 892.73 | 0.79 | 0.43 | -0.04 | 0.08 | 0.03 |
| Status |  |  |  |  |  |  |  |  |
| Intercept for negative difference | 4.21 | 0.03 | 429.45 | 152.93 | 0.00 | 4.16 | 4.26 | - |
| Intercept for positive difference | 4.20 | 0.04 | 753.61 | 119.22 | 0.00 | 4.13 | 4.27 | - |
| Actor negative difference | 0.06 | 0.03 | 1002.23 | 2.25 | 0.02 | 0.01 | 0.12 | 0.07 |
| Actor positive difference | 0.05 | 0.04 | 851.90 | 1.16 | 0.25 | -0.03 | 0.12 | 0.04 |
| Partner negative difference | 0.04 | 0.03 | 1003.93 | 1.62 | 0.11 | -0.01 | 0.09 | 0.05 |
| Partner positive difference | 0.06 | 0.03 | 886.70 | 1.92 | 0.05 | 0.00 | 0.11 | 0.06 |

| *Estimates in the Over-Time Standard APIM for Mean-Level Match's Effect on Relationship Satisfaction* | | | | | | | |  |
| --- | --- | --- | --- | --- | --- | --- | --- | --- |
|  |  |  |  |  |  | 95% CI | | |
|  | Estimate | *SE* | *df* | *t* | *p* | Lower bound | Upper bound | *r* |
| Warmth |  |  |  |  |  |  |  |  |
| Intercept for negative difference | 111.57 | 0.67 | 343.23 | 167.25 | 0.00 | 110.26 | 112.89 | - |
| Intercept for positive difference | 111.67 | 0.85 | 664.82 | 132.06 | 0.00 | 110.01 | 113.33 | - |
| Actor negative difference | 4.24 | 0.79 | 937.79 | 5.34 | 0.00 | 2.68 | 5.80 | 0.17 |
| Actor positive difference | -0.36 | 0.97 | 840.77 | -0.37 | 0.71 | -2.27 | 1.55 | 0.01 |
| Partner negative difference | 1.09 | 0.77 | 947.58 | 1.41 | 0.16 | -0.42 | 2.59 | 0.05 |
| Partner positive difference | 1.06 | 0.73 | 865.25 | 1.45 | 0.15 | -0.37 | 2.49 | 0.05 |
| Vitality |  |  |  |  |  |  |  |  |
| Intercept for negative difference | 112.26 | 0.68 | 371.25 | 165.41 | 0.00 | 110.92 | 113.59 | - |
| Intercept for positive difference | 112.18 | 0.85 | 693.33 | 131.75 | 0.00 | 110.51 | 113.85 | - |
| Actor negative difference | 3.72 | 0.55 | 939.31 | 6.77 | 0.00 | 2.64 | 4.80 | 0.22 |
| Actor positive difference | -0.09 | 0.91 | 806.20 | -0.10 | 0.92 | -1.88 | 1.70 | 0.00 |
| Partner negative difference | 0.85 | 0.52 | 941.80 | 1.63 | 0.10 | -0.17 | 1.88 | 0.05 |
| Partner positive difference | -0.02 | 0.68 | 870.04 | -0.03 | 0.97 | -1.37 | 1.32 | 0.00 |
| Status |  |  |  |  |  |  |  |  |
| Intercept for negative difference | 111.94 | 0.69 | 378.35 | 161.57 | 0.00 | 110.57 | 113.30 | - |
| Intercept for positive difference | 111.60 | 0.85 | 664.75 | 131.01 | 0.00 | 109.93 | 113.27 | - |
| Actor negative difference | 2.50 | 0.63 | 963.67 | 3.99 | 0.00 | 1.27 | 3.73 | 0.13 |
| Actor positive difference | 0.49 | 0.88 | 830.08 | 0.56 | 0.58 | -1.24 | 2.23 | 0.02 |
| Partner negative difference | 1.75 | 0.59 | 968.40 | 2.94 | 0.00 | 0.58 | 2.91 | 0.09 |
| Partner positive difference | 0.03 | 0.65 | 862.04 | 0.05 | 0.96 | -1.25 | 1.32 | 0.00 |

|  |  |  |  |  |  |  |  |  |  |
| --- | --- | --- | --- | --- | --- | --- | --- | --- | --- |
| *Estimates in the Over-Time Standard APIM of Acceptance Using Pattern Correspondence and Controlling for Gender* | | | | | | | | |  |
|  |  |  |  |  |  | 95% CI | | |  |
|  | Estimate | *SE* | *df* | *t* | *p* | Lower bound | Upper bound | *r* | |
| Linear Model |  |  |  |  |  |  |  |  | |
| Intercept | 4.21 | 0.02 | 175.42 | 194.24 | 0.00 | 4.16 | 4.25 | - | |
| Actor Consistency (Linear) | 0.06 | 0.04 | 783.03 | 1.50 | 0.13 | -0.02 | 0.15 | 0.05 | |
| Partner Consistency (Linear) | 0.05 | 0.04 | 785.41 | 1.26 | 0.21 | -0.03 | 0.14 | 0.04 | |
| Gender | 0.04 | 0.04 | 184.66 | 1.07 | 0.29 | -0.03 | 0.11 | 0.08 | |
| Negative Exponential Model |  |  |  |  |  |  |  |  | |
| Intercept | 4.34 | 0.06 | 526.39 | 70.44 | 0.00 | 4.21 | 4.46 | - | |
| Actor Consistency (Exponential) | -0.06 | 0.04 | 777.77 | -1.75 | 0.08 | -0.14 | 0.01 | 0.06 | |
| Partner Consistency (Exponential) | -0.06 | 0.04 | 779.43 | -1.62 | 0.11 | -0.13 | 0.01 | 0.06 | |
| Gender | 0.04 | 0.04 | 184.57 | 1.07 | 0.28 | -0.03 | 0.11 | 0.08 | |

| *Estimates in the Over-Time Standard APIM of Relationship Satisfaction Using Pattern Correspondence and Controlling for Gender* | | | | | | | | |
| --- | --- | --- | --- | --- | --- | --- | --- | --- |
|  |  |  |  |  |  | 95% CI | | |
|  | Estimate | *SE* | *df* | *t* | *p* | Lower bound | Upper bound | *r* |
| Linear Model |  |  |  |  |  |  |  |  |
| Intercept | 110.48 | 0.62 | 172.09 | 178.98 | 0.00 | 109.26 | 111.70 | - |
| Actor Consistency (Linear) | 2.59 | 0.98 | 723.30 | 2.64 | 0.01 | 0.67 | 4.52 | 0.10 |
| Partner Consistency (Linear) | 0.70 | 0.98 | 747.86 | 0.72 | 0.47 | -1.22 | 2.63 | 0.03 |
| Gender | -0.52 | 0.76 | 183.94 | -0.68 | 0.50 | -2.01 | 0.98 | 0.05 |
| Negative Exponential Model |  |  |  |  |  |  |  |  |
| Intercept | 113.52 | 1.49 | 465.82 | 76.10 | 0.00 | 110.59 | 116.45 | - |
| Actor Consistency (Exponential) | -2.42 | 0.86 | 723.06 | -2.82 | 0.00 | -4.11 | -0.74 | 0.10 |
| Partner Consistency (Exponential) | -0.48 | 0.86 | 739.07 | -0.56 | 0.58 | -2.16 | 1.21 | 0.02 |
| Gender | -0.53 | 0.76 | 183.77 | -0.70 | 0.48 | -2.03 | 0.96 | 0.05 |

| *Estimates in the Over-Time Standard APIM of Acceptance Using Mean-Level Match and Controlling for Gender* | | | | | | | | |
| --- | --- | --- | --- | --- | --- | --- | --- | --- |
|  |  |  |  |  |  | 95% CI | | |
|  | Estimate | *SE* | *df* | *t* | *p* | Lower bound | Upper bound | *r* |
| Intercept for negative difference | 4.22 | 0.03 | 480.00 | 151.25 | 0.00 | 4.17 | 4.28 | - |
| Intercept for positive difference | 4.21 | 0.03 | 675.07 | 130.87 | 0.00 | 4.14 | 4.27 | - |
| Actor negative difference | 0.18 | 0.04 | 988.44 | 4.48 | 0.00 | 0.10 | 0.26 | 0.14 |
| Actor positive difference | 0.09 | 0.05 | 855.09 | 1.62 | 0.11 | -0.02 | 0.19 | 0.06 |
| Partner negative difference | 0.02 | 0.04 | 997.88 | 0.62 | 0.54 | -0.05 | 0.09 | 0.02 |
| Partner positive difference | 0.09 | 0.04 | 881.98 | 2.07 | 0.04 | 0.00 | 0.18 | 0.07 |
| Gender | 0.04 | 0.03 | 193.91 | 1.04 | 0.30 | -0.03 | 0.10 | 0.07 |

| *Estimates in the Over-Time Standard APIM of Relationship Satisfaction Using Mean-Level Match and Controlling for Gender* | | | | | | | | | |
| --- | --- | --- | --- | --- | --- | --- | --- | --- | --- |
|  |  |  |  |  |  | 95% CI | | |  |
|  | Estimate | *SE* | *df* | *t* | *p* | Lower bound | Upper bound | *r* | |
| Intercept for negative difference | 112.54 | 0.69 | 417.82 | 163.52 | 0.00 | 111.19 | 113.89 | - |  |
| Intercept for positive difference | 112.83 | 0.78 | 588.18 | 145.41 | 0.00 | 111.31 | 114.35 | - |  |
| Actor negative difference | 5.46 | 0.90 | 966.30 | 6.07 | 0.00 | 3.70 | 7.23 | 0.19 |  |
| Actor positive difference | -0.27 | 1.22 | 834.35 | -0.22 | 0.82 | -2.68 | 2.13 | 0.01 |  |
| Partner negative difference | 2.73 | 0.83 | 973.24 | 3.29 | 0.00 | 1.10 | 4.37 | 0.10 |  |
| Partner positive difference | -0.82 | 0.98 | 861.54 | -0.83 | 0.40 | -2.74 | 1.11 | 0.03 |  |
| Gender | -0.98 | 0.72 | 188.82 | -1.36 | 0.18 | -2.41 | 0.44 | 0.10 |  |

| *Estimates in the Over-Time Standard APIM of Acceptance Using Pattern Correspondence and Checking for Gender Interaction* | | | | | | | | |
| --- | --- | --- | --- | --- | --- | --- | --- | --- |
|  |  |  |  |  |  | 95% CI | | |
|  | Estimate | *SE* | *df* | *t* | *p* | Lower bound | Upper bound | *r* |
| Linear Model |  |  |  |  |  |  |  |  |
| Intercept | 4.21 | 0.02 | 178.03 | 193.42 | 0.00 | 4.16 | 4.25 | - |
| Actor Consistency (Linear) | 0.06 | 0.04 | 782.72 | 1.50 | 0.14 | -0.02 | 0.15 | 0.05 |
| Partner Consistency (Linear) | 0.05 | 0.04 | 784.69 | 1.26 | 0.21 | -0.03 | 0.14 | 0.04 |
| Gender | 0.04 | 0.04 | 184.57 | 1.07 | 0.29 | -0.03 | 0.11 | 0.08 |
| Actor Consistency (Linear) X Gender | -0.04 | 0.08 | 783.43 | -0.46 | 0.65 | -0.20 | 0.13 | 0.02 |
| Partner Consistency (Linear) X Gender | -0.01 | 0.08 | 779.93 | -0.12 | 0.91 | -0.17 | 0.15 | 0.00 |
| Negative Exponential Model |  |  |  |  |  |  |  |  |
| Intercept | 4.34 | 0.06 | 526.07 | 70.35 | 0.00 | 4.21 | 4.46 | - |
| Actor Consistency (Exponential) | -0.06 | 0.04 | 777.71 | -1.71 | 0.09 | -0.14 | 0.01 | 0.06 |
| Partner Consistency (Exponential) | -0.06 | 0.04 | 778.07 | -1.62 | 0.11 | -0.13 | 0.01 | 0.06 |
| Gender | -0.01 | 0.11 | 502.01 | -0.06 | 0.95 | -0.23 | 0.21 | 0.00 |
| Actor Consistency (Exponential) X Gender | 0.03 | 0.07 | 777.91 | 0.47 | 0.64 | -0.11 | 0.18 | 0.02 |
| Partner Consistency (Exponential) X Gender | 0.01 | 0.07 | 774.56 | 0.15 | 0.88 | -0.13 | 0.16 | 0.01 |

| *Estimates in the Over-Time Standard APIM of Relationship Satisfaction Using Pattern Correspondence and Checking for Gender Interaction* | | | | | | | | | |
| --- | --- | --- | --- | --- | --- | --- | --- | --- | --- |
|  |  |  |  |  |  | 95% CI | | |  |
|  | Estimate | *SE* | *df* | *t* | *p* | Lower bound | Upper bound | *r* |  |
| Linear Model |  |  |  |  |  |  |  |  |  |
| Intercept | 110.52 | 0.62 | 173.64 | 178.87 | 0.00 | 109.30 | 111.74 | - |  |
| Actor Consistency (Linear) | 2.60 | 0.98 | 723.38 | 2.65 | 0.01 | 0.67 | 4.53 | 0.10 |  |
| Partner Consistency (Linear) | 0.67 | 0.98 | 747.44 | 0.68 | 0.50 | -1.26 | 2.60 | 0.02 |  |
| Gender | -0.53 | 0.76 | 184.68 | -0.70 | 0.49 | -2.03 | 0.97 | 0.05 |  |
| Actor Consistency (Linear) X Gender | 0.30 | 1.93 | 742.28 | 0.16 | 0.88 | -3.48 | 4.08 | 0.01 |  |
| Partner Consistency (Linear) X Gender | 2.48 | 1.93 | 719.61 | 1.29 | 0.20 | -1.30 | 6.26 | 0.05 |  |
| Negative Exponential Model |  |  |  |  |  |  |  |  |  |
| Intercept | 113.44 | 1.50 | 467.16 | 75.85 | 0.00 | 110.51 | 116.38 | - |  |
| Actor Consistency (Exponential) | -2.43 | 0.86 | 723.95 | -2.82 | 0.00 | -4.12 | -0.74 | 0.10 |  |
| Partner Consistency (Exponential) | -0.35 | 0.86 | 738.37 | -0.41 | 0.68 | -2.04 | 1.34 | 0.02 |  |
| Gender | 2.60 | 2.49 | 474.65 | 1.04 | 0.30 | -2.30 | 7.51 | 0.05 |  |
| Actor Consistency (Exponential) X Gender | -0.29 | 1.69 | 736.21 | -0.17 | 0.86 | -3.61 | 3.03 | 0.01 |  |
| Partner Consistency (Exponential) X Gender | -2.73 | 1.69 | 718.66 | -1.61 | 0.11 | -6.05 | 0.60 | 0.06 |  |

| *Estimates in the Over-Time Standard APIM of Acceptance Using Mean-Level Match and Checking for Gender Interaction* | | | | | | | | |
| --- | --- | --- | --- | --- | --- | --- | --- | --- |
|  |  |  |  |  |  | 95% CI | | |
|  | Estimate | *SE* | *df* | *t* | *p* | Lower bound | Upper bound | *r* |
| Intercept for negative difference | 4.22 | 0.03 | 480.12 | 151.08 | 0.00 | 4.17 | 4.28 | - |
| Intercept for positive difference | 4.21 | 0.03 | 673.64 | 130.79 | 0.00 | 4.14 | 4.27 | - |
| Actor negative difference | 0.18 | 0.04 | 988.11 | 4.48 | 0.00 | 0.10 | 0.26 | 0.14 |
| Actor positive difference | 0.09 | 0.05 | 854.01 | 1.61 | 0.11 | -0.02 | 0.19 | 0.06 |
| Partner negative difference | 0.02 | 0.04 | 998.76 | 0.61 | 0.54 | -0.05 | 0.09 | 0.02 |
| Partner positive difference | 0.09 | 0.04 | 878.42 | 2.08 | 0.04 | 0.00 | 0.18 | 0.07 |
| Gender | 0.05 | 0.05 | 406.14 | 1.14 | 0.26 | -0.04 | 0.14 | 0.06 |
| Actor negative difference X Gender | 0.07 | 0.07 | 979.87 | 0.93 | 0.35 | -0.08 | 0.21 | 0.03 |
| Actor positive difference X Gender | 0.03 | 0.09 | 879.76 | 0.35 | 0.73 | -0.14 | 0.20 | 0.01 |
| Partner negative difference X Gender | 0.01 | 0.07 | 985.01 | 0.10 | 0.92 | -0.14 | 0.15 | 0.00 |
| Partner positive difference X Gender | -0.03 | 0.09 | 867.55 | -0.37 | 0.71 | -0.20 | 0.14 | 0.01 |

| *Estimates in the Over-Time Standard APIM of Relation Satisfaction Using Mean-Level Match and Checking for Gender Interaction* | | | | | | | | |
| --- | --- | --- | --- | --- | --- | --- | --- | --- |
|  |  |  |  |  |  | 95% CI | | |
|  | Estimate | *SE* | *df* | *t* | *p* | Lower bound | Upper bound | *r* |
| Intercept for negative difference | 112.55 | 0.69 | 417.21 | 163.64 | 0.00 | 111.20 | 113.90 | - |
| Intercept for positive difference | 112.80 | 0.78 | 587.08 | 145.40 | 0.00 | 111.28 | 114.32 | - |
| Actor negative difference | 5.43 | 0.90 | 965.30 | 6.04 | 0.00 | 3.67 | 7.19 | 0.19 |
| Actor positive difference | -0.21 | 1.22 | 833.48 | -0.17 | 0.86 | -2.61 | 2.19 | 0.01 |
| Partner negative difference | 2.78 | 0.83 | 973.85 | 3.35 | 0.00 | 1.15 | 4.41 | 0.11 |
| Partner positive difference | -0.86 | 0.98 | 859.34 | -0.88 | 0.38 | -2.79 | 1.06 | 0.03 |
| Gender | -2.14 | 1.00 | 408.51 | -2.15 | 0.03 | -4.11 | -0.18 | 0.11 |
| Actor negative difference X Gender | -1.71 | 1.67 | 918.85 | -1.02 | 0.31 | -4.99 | 1.57 | 0.03 |
| Actor positive difference X Gender | 0.55 | 1.97 | 848.02 | 0.28 | 0.78 | -3.32 | 4.41 | 0.01 |
| Partner negative difference X Gender | -1.88 | 1.67 | 935.53 | -1.12 | 0.26 | -5.16 | 1.41 | 0.04 |
| Partner positive difference X Gender | 1.99 | 1.97 | 826.13 | 1.01 | 0.31 | -1.87 | 5.86 | 0.04 |
